# Too much information? Males convey parasite levels using more signal modalities than females utilise

**DOI:** 10.1242/jeb.246217

**Published:** 2024-01-10

**Authors:** Arka Pal, Mihir Joshi, Maria Thaker

**Affiliations:** ^1^ Centre for Ecological Sciences, Indian Institute of Science, Bangalore 560 012, India; ^2^ Institute of Science and Technology Austria, 3400 Klosterneuburg, Austria

**Keywords:** Animal communication, Parasites, Sexual selection, Visual, Chemical, Mate choice, Hamilton–Zuk hypothesis

## Abstract

Elaborate sexual signals are thought to have evolved and be maintained to serve as honest indicators of signaller quality. One measure of quality is health, which can be affected by parasite infection. *Cnemaspis mysoriensis* is a diurnal gecko that is often infested with ectoparasites in the wild, and males of this species express visual (coloured gular patches) and chemical (femoral gland secretions) traits that receivers could assess during social interactions. In this paper, we tested whether ectoparasites affect individual health, and whether signal quality is an indicator of ectoparasite levels. In wild lizards, we found that ectoparasite level was negatively correlated with body condition in both sexes. Moreover, some characteristics of both visual and chemical traits in males were strongly associated with ectoparasite levels. Specifically, males with higher ectoparasite levels had yellow gular patches with lower brightness and chroma, and chemical secretions with a lower proportion of aromatic compounds. We then determined whether ectoparasite levels in males influence female behaviour. Using sequential choice trials, wherein females were provided with either the visual or the chemical signals of wild-caught males that varied in ectoparasite level, we found that only chemical secretions evoked an elevated female response towards less parasitised males. Simultaneous choice trials in which females were exposed to the chemical secretions from males that varied in parasite level further confirmed a preference for males with lower parasites loads. Overall, we find that although health (body condition) or ectoparasite load can be honestly advertised through multiple modalities, the parasite-mediated female response is exclusively driven by chemical signals.

## Teaser

**Summary:** In a diurnal gecko species, ectoparasite level and overall health are honestly advertised through multiple modalities, yet females assess males based only on their chemical signals, raising questions about redundancy in signals that are not used.

## INTRODUCTION

The evolution and maintenance of elaborate traits in species is widely attributed to sexual selection, with many hypotheses arguing that mates are selected based on traits linked to individual quality (Andersson, 1994; Zahavi, 1975), while other arguments include runaway selection, sexual conflict or intra-sexual selection. If mate selection is based on individual quality, then two aspects of signalling traits are especially important: information content and the degree of honesty associated with those signals. Honesty of sexual signals can be maintained through mechanisms that associate the signals and their costs with the individual's quality (Biernaskie et al., 2014; Zahavi, 1975). When signals are complex or involve multiple components, individual traits can either signal different measures of individual quality (‘multiple-messages’) or reliably reinforce the individual quality (‘back-up signals’) (Johnstone, 1997).

One important measure of quality in social interactions is parasite resistance (Hamilton and Zuk, 1982). Traits that encode information about parasite load have direct and indirect benefits for potential mates during social communication. These traits could indicate the risk of parasite transmission to the receiver, as well as the overall health of the signaller (Weaver et al., 2017) and the quality of its immune system (Folstad and Karter, 1992). Since its inception, parasite-mediated sexual signalling (i.e. Hamilton–Zuk hypothesis) has been hotly debated, with mixed empirical support (Balenger and Zuk, 2014). There is considerable evidence for an association between parasite load and the quality of secondary sexual signals (del Cerro et al., 2010; Martínez-Padilla et al., 2007), driven by the cost of parasitism on health and/or survival probability (Sánchez et al., 2018). Studies across several taxa have also reported mate choice based on traits that may indicate parasite resistance (Rantala and Kortet, 2003; Stephenson et al., 2020). However, this is not a consistent trend as there is also evidence of no correlation between the quality of elaborate traits and parasite load (Setchell et al., 2009; Sorensen et al., 2016), or between parasite load and mate preference (Aguilar et al., 2008). Although proximate mechanisms of how health affects secondary sexual traits are now better understood (Hill, 2011), evidence for potential mates scrutinising such ‘honest’ traits of parasite resistance is inconsistent.

Similar to that in other taxonomic groups, parasite-mediated sexual selection in lizards has also had mixed support. Many lizards express sexually dimorphic visual (Kabir et al., 2019) and chemical traits (Martín and López, 2015) in isolation or together (Kabir et al., 2020). These traits can reflect individual quality, enabling them to be used as effective signals in intrasexual competition (Olsson et al., 2013) and/or intersexual selection (Bajer et al., 2010; López and Martín, 2005). There is growing evidence that elaborate visual signals, such as coloration in lizards, are influenced by parasite load (Badiane et al., 2022; Martín and López, 2009; Megía-Palma et al., 2016). Similarly, chemical secretions are often good indicators of male health status or parasite load in lizards (López et al., 2006; Martín et al., 2007). When species express multiple signals across more than one modality, it is not clear whether information about parasite load or health is encoded in one or more modalities (Martín et al., 2008). For conspecifics that are assessing these signals, obtaining reliable information is important. Studying both signallers and receivers in more species that express traits in multiple modalities would provide a broader understanding of the relevance of multimodality in parasite-mediated sexual signalling.

The Mysore day gecko (*Cnemaspis mysoriensis*) is a small endemic species found in the Bangalore–Mysore region of southern India (Giri et al., 2009; Jerdon, 1853). These geckos are reproductively active throughout the year, as evidenced by patterns of cohabitation, mating and egg laying (Biswas, 2005; Kabir, 2021; Kabir et al., 2019). Individuals of this species are often infected by ectoparasites of *Geckobia* sp. (Acariformes: Pterygosomatidae) (Fajfer and Karanth, 2021). Males express potential information-containing signalling traits in two modalities. On the one hand, they have colour-based visual traits that females lack – yellow eye rims, and also a yellow patch on their gular in some cases (Kabir et al., 2019). On the other hand, they have femoral and pre-cloacal glands that deposit chemical secretions on the ventral surface (Giri et al., 2009). Secretions from these glands contain a complex mix of molecules, including saturated fatty acids, such as hexadecanoic acid, and aromatic compounds, such as benzoic acid, in addition to cholesterol and squalene that are found only in male secretions (Table S2). The chemical composition of these ventral secretions is similar in all males regardless of whether their gular has a yellow patch or not (Kabir et al., 2019). In this species, chemical secretions, but not the presence or absence of the yellow gular patch of males, are necessary to elicit a female response and are effective signals of sex identity (Kabir et al., 2019, 2020). Unlike females, males use both the chemical and visual traits of other males during social interactions (Kabir et al., 2019).

Here, we posit that ectoparasites may influence signals and receiver responses in *C. mysoriensis*. We first explored the association between ectoparasite levels and body condition in wild lizards of both sexes, testing the hypothesis that ectoparasite levels are negatively associated with this health indicator. We then assessed the effect of ectoparasites on both the chemical and visual components of male sexual signals: yellow gular coloration and composition of ventral secretions. If investment in immunity against parasites is energetically expensive and resources are drawn away from sexual signal expression (Folstad and Karter, 1992), we predict a negative correlation between ectoparasite level and the intensity (chroma and/or brightness) of the yellow gular patch, and the complexity of composition of the chemical secretions. Finally, we tested whether ectoparasite load affects female responses towards a male conspecific, using sequential and simultaneous behavioural trials with wild-caught animals that naturally vary in infection level. If females can discriminate between males with different ectoparasite load using their visual or chemical traits, we would expect a preference towards non-parasitized males. This series of experiments allowed us not only to examine the effect of ectoparasite levels on male signals in multiple modalities but also to reveal the consequences of this in terms of the female response towards males, providing insight into the evolution and maintenance of multimodal signals.

## MATERIALS AND METHODS

Animal ethics approvals were obtained from the Institutional Animal Ethics Committee of IISc (CAF/Ethics/489/2016).

### Ectoparasite level and body condition

To capture the variation in parasite load and body size metrics, we sampled *Cnemaspis mysoriensis* in the forested areas of the Indian Institute of Science (IISc) campus (Bangalore) every month from September 2015 to March 2016. Sampling was carried out in plots (*n*=6) of 25 m^2^, that were at least 200 m apart from each other. Previous field observations suggest almost no dispersal or movement between plots that are at least 200 m apart. Plots had a density of 27–52 individuals per plot. We captured females and only males with yellow gular patches because they are the more abundant morph (field observation) and express both visual and chemical traits (Kabir et al., 2019). Individuals from the field (*n*=392; males *n*=212; females *n*=180) were brought to the laboratory and kept in plastic containers lined with clean paper towels (30×20×10 cm^3^) in a dedicated housing space with windows that allowed natural lighting conditions (average temperature of the room 26±2°C) (Joshi et al., 2022). During their stay in the housing area, individuals had access to water and *Drosophila* flies *ad libitum*. For each individual, we measured body mass (using a digital weighing balance; Sartorius GE412), snout–vent length (SVL; using vernier callipers; Aerospace) and ectoparasite level. Ectoparasites of *Geckobia* sp were counted on the body surface of each lizard, using a magnifying glass. These ectoparasites cluster around the throat and appendages (A.P., M.J. and M.T., unpublished observation). No other ectoparasites (e.g. ticks) were found on these lizards. After measurement, each individual was marked on its ventral surface with a non-toxic marker pen and released at the site of capture within 8 h.

We used body mass and SVL to calculate the scaled mass index (SMI) as a measure of body condition separately for males and females (Peig and Green, 2010). The distribution of ectoparasite level across individuals was zero-inflated (‘performance’ package in R; http://www.R-project.org/) and non-normally distributed (Shapiro–Wilk test; *P*<0.001) (Fig. S1). Therefore, to model variation in ectoparasite level, we performed a Poisson generalised linear mixed model (GLMM) using the ‘glmmADMB’ package (Fournier et al., 2012) in R. We included sex, sampling month (September to March) and SMI, including the interaction between SMI and sex as fixed factors and sampling plot as random factor.

### Measuring visual signals in males

Visual signals were obtained from 40 males sampled in September 2015. Each individual was brought to the laboratory and kept in the same plastic containers (30×20×10 cm^3^) in the dedicated housing space as described above, with access to water and *Drosophila* flies *ad libitum*, until measurements were taken. In addition to measuring body mass and SVL, and counting ectoparasites, we photographed individuals using a Canon EOS 700-D DSLR camera in the lab with a camera flash on a neutral grey cardstock board that ensured that images were not over- or under-exposed. Every image included an X-Rite Color Checker. Photographed lizards were marked on their ventral side with a permanent non-toxic marker and were released at the site of capture within 8 h.

Following the approach established by Stevens et al. (2007), we quantified hue, chroma and brightness from these digital photographs. The yellow gular patch in males is not UV reflective (Kabir et al., 2019) and thus digital imagery was the best way to obtain colour traits of the entire patch. First, mean red–green–blue (RGB) and brightness values from manually outlined gular regions of the males were linearized and equalized with respect to the reflectance of the six greyscale squares of the X-Rite Color Checker standard. Brightness was quantified by summing up R, G and B values. Relative brightness contrasts between red–green (RG) and green–blue (GB) were calculated as follows: RG=(R−G)/brightness and GB=(G−B)/brightness. Finally, hue was measured as arctan(GB/RG) (in radians) and chroma as (RG^2^+GB^2^)^0.5^ (Endler, 1990; Grill and Rush, 2000). Extractions for all colour parameters were done in MATLAB (R2019a, The MathWorks, Inc., Natick, MA, USA). To model the variation in visual signals with respect to ectoparasite levels, we performed separate GLMMs with brightness, chroma and hue as response variables, and ectoparasite level, SMI and the interaction between them as fixed factors. Finally, plot (location of capture) was included as a random effect. Statistical analyses were run using the ‘lme4’ package in R.

### Measuring chemical signals in males

For the collection of chemical secretions, we sampled 30 males with yellow gulars from various locations on the IISc campus between February and March 2021. Similar to the above, we measured each individual for their parasite level, body mass and SVL. Each wild-caught individual was housed in a separate plastic container (30×20×10 cm^3^) in the same housing space described above; during captivity, individuals were provided with *Drosophila* flies *ad libitum* for food and water. Animals were allowed to acclimate to the housing conditions for 24 h, after which the femoral pore and pre-cloacal secretions of each individual were collected for 10 consecutive days and pooled in a single vial. To collect these chemical secretions, we gently scraped the thin film on the ventral surface of each male using a sterilised metal spatula and dissolved them in 1 ml dichloromethane (Merck, HPLC Grade). Chemical secretions were stored in −20°C until analysis. After a total of 11 days in captivity, the individuals were released at the site of capture.

Chemical secretions were analysed by gas chromatography – mass spectrometry (GCMS) using Agilent 7000D/GCTQ. We followed the GCMS analysis protocol as described in Kabir et al. (2019). From the chromatograms, we measured the relative amounts of all compounds as the percentage of total ion current (%TIC). Three parameters of chemical signals were measured: (1) the number of compounds present in the secretions (chemical richness); (2) the relative proportion of aromatic compounds; and (3) the relative proportion of saturated compounds. We modelled the variation in proportion of each chemical signal with respect to ectoparasite levels by performing separate GLMMs with each chemical compound as the response variable. In these models, ectoparasite level, SMI and their interaction were fixed factors, whereas capture location of individuals was a random effect.

### Female response trials – sequential presentation of male stimuli in different signaling modalities

To determine whether females can distinguish between males with different ectoparasite levels based on visual and chemical stimuli only, we conducted a sequential presentation experiment in the laboratory using wild-caught unmanipulated lizards. Focal females (*n*=30) and stimulus males (*n*=6) were captured in February and March 2016 from different sampling plots on the IISc campus to ensure no previous encounters. Stimulus males were either parasitized (*n*=3 males with 8–10 ectoparasites per individual; hereafter, P males) or non-parasitized (*n*=3; hereafter, NP males). All other morphological measurements of the stimulus males were similar (SVL 28±2 mm, body mass 0.5±0.05 g for NP males and 0.35±0.05 g for P males). Captured individuals were brought into the lab and acclimatised for 5 days in individual plastic housing containers (30×20×10 cm^3^) with access to water and *Drosophila* flies *ad libitum*, following the same housing conditions described above.

Each trial consisted of a female being exposed sequentially to the potential signalling traits of two males. For testing female responses separately to visual and chemical traits, the trials were grouped into three treatment types – control 1 (both males were NP), control 2 (both males were P) and test (one male was P while the other was NP, sequentially presented to females in randomised order) (Fig. S2). The controls were necessary to determine female responses towards the traits of two different but morphologically similar males (P or NP), with the expectation that female responses would be similar. The pairs of males used within each treatment (control 1, control 2 and test) and for each signalling modality (chemical or visual) were kept constant to exclude any other potential unmeasurable variation. In other words, females were exposed to the chemical and visual traits of the same pair of males in each experiment.

For chemical stimulus-only trials, we used paper towels that predominantly contained the depositions of male pre-cloacal and femoral pore secretions as the stimulus. These paper towels lined the housing containers of stimulus males for 2 days immediately before use in the female-response trials. Faecal matter, if any, was removed every day. For the visual stimulus-only trials, we used live males that were wiped clean with dampened cotton and subsequently prevented from secreting chemicals by physically blocking their pre-cloacal and femoral glands with petroleum jelly (López et al., 2003). Exposure to visual stimuli (gular coloration) was impossible to conduct in isolation of the signaller as artificial lizard models were ineffective in eliciting a response from females (A.P., M.J. and M.T., personal observations).

All behavioural trials were carried out in a separate sanitised experimental arena (30×20×10 cm^3^) in the lab between 08:00 h and 15:00 h when animals were fully active. Each focal female was individually introduced into the trial tanks and given 10 min to habituate inside refuge cups of 10 cm diameter. Once the refuge cup was removed, we recorded the behaviour of females towards the visual or chemical stimulus, which was placed inside the arena for 10 min. After the first stimulus exposure, females were removed from the testing tank and given 30 min without disturbance. We then cleaned the testing tank with 50% ethanol and introduced the second stimulus. The behavioural trial was then repeated with the female reintroduced into the tank and allowed a 10 min habituation period, followed by a 10 min interaction period. Each control (NP versus NP or P versus P) and the test (NP versus P) was performed on different days separated by a day to minimise fatigue and habituation.

Female responses during the trials were measured as the number of tongue flicks and number of movement bouts. Tongue flicks included any extrusion of the tongue into air or onto the substrate. A movement bout was defined as the displacement of the focal animal involving at least three steps defined as complete locomotor cycles. Two successive tongue flicks or movement bouts were defined when separated by at least 3 s of no activity. Both tongue flicks and movement bouts have previously been used as measures for female responses towards both visual and chemical stimuli in this and related species (Kabir and Thaker, 2021; Kabir et al., 2019; Raya-García et al., 2020).

We calculated relative female response as the difference in the number of tongue flicks or movement bouts to the male stimuli in the sequential exposure. Relative difference was calculated for each treatment type and sensory modality separately, and the order of subtraction was kept constant based on male ID (NP1−NP2 for control 1, P1−P2 for control 2, NP−P for test). A relative response value of 0 in any trial suggests that females had no differential response towards either stimulus, whereas a positive response value in the test treatment suggests that the female was more responsive towards the NP male than to the P male, and the inverse in the case of a negative response value. Within each sensory modality, we modelled the variation in relative female responses with a Poisson GLMM (‘glmmADMB’ statistical package in R), with treatment type (control 1, control 2, test) as a fixed factor and female ID as the random factor. We then tested pairwise significance between treatment types using the ‘multcomp’ package in R.

### Female choice trials – simultaneous presentation of male stimuli

To further determine the effect of ectoparasites on female choice, females were offered a simultaneous choice between a low parasitised (<4 ectoparasites; hereafter, LP males) and a high parasitised (>10 ectoparasites; hereafter, HP males) male in a Y-tube setup (Fig. S2). For this experiment, we collected focal females (*n*=30) and stimulus males from separate locations on the IISc campus between January and February 2021 to avoid the effect of previous encounters. All other morphological aspects of the stimulus males were within normal range (SVL 2.5–3 cm, body mass 0.45–0.62 g). Each individual was housed in a separate container for 7 days in total under the same housing conditions as described above. Female choice experiments were carried out after 24 h of acclimation.

For the control trials, females were given a choice between 2 LP males or 2 HP males. As opposed to the sequential response experiment in which the stimuli were from males that either harboured or did not harbour mites, this simultaneous choice trial was designed to determine whether females can comparatively distinguish between the chemical stimuli of males that differed in ectoparasite level (low versus high). For this experiment, only chemical signals were used because in the sequential response experiment described above, females responded only to chemical stimuli. In all the choice combinations, thin strips of paper towels with male chemical secretions (see description above) were lined along both arms of the Y-tube setup such that the females could detect the secretions of both the males simultaneously. Each stimulus strip was randomly assigned to one of the sides of the setup to minimise the potential effect of side bias (Fig. S2).

Focal females were given a 10 min habituation period under a refuge cup at the entrance of the Y-tube, followed by a 20 min recording of their response when released. Choice was determined only when the females completely entered one of the Y-tube arms. If the female did not choose either of the males, the trial was recorded as ‘no choice’. The Y-tube setup was cleaned with 50% ethanol and water between trials to eliminate any traces from the previous trials. All trials were video recorded and were designed to be ‘double-blind’, such that the experimenter was unaware of the stimulus male identity. If females choose males based on their chemical secretions, the proportion of total choices towards LP males should be significantly greater than 0.5. To test this prediction, we carried out Fisher's exact test of proportions separately for all three treatments, i.e. high versus low, high versus high, and low versus low.

## RESULTS

### Relationship of ectoparasite level to body condition and sex

**Fig. 1.**
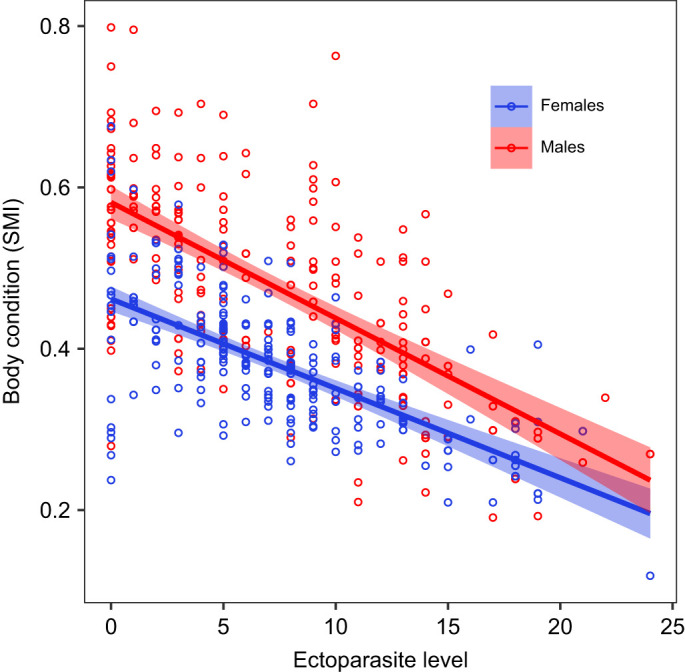
**Relationship between ectoparasite level and body condition.** Lizards with lower ectoparasite level (number of parasites) had a higher body condition (measured as scaled mass index, SMI) (*z*=−5.26, *P*<0.001). Lines indicate the generalised linear mixed model (GLMM) fit to each sex, and the shaded area constitutes the 95% confidence interval. *n*=212 males and *n*=180 females.

Ectoparasite levels on lizards varied as a function of body condition, sex and sampling month (Fig. 1; Fig. S1, Table S1). Overall, individuals with a higher number of ectoparasites had poorer body condition (*z*=−5.26, *P*<0.001; Fig. 1), and this relationship was stronger in males than in females (interaction between sex and body condition: *z=*1.62, *P*<0.001; Fig. 1).

### Relationship between ectoparasite level and visual signal

**Fig. 2.**
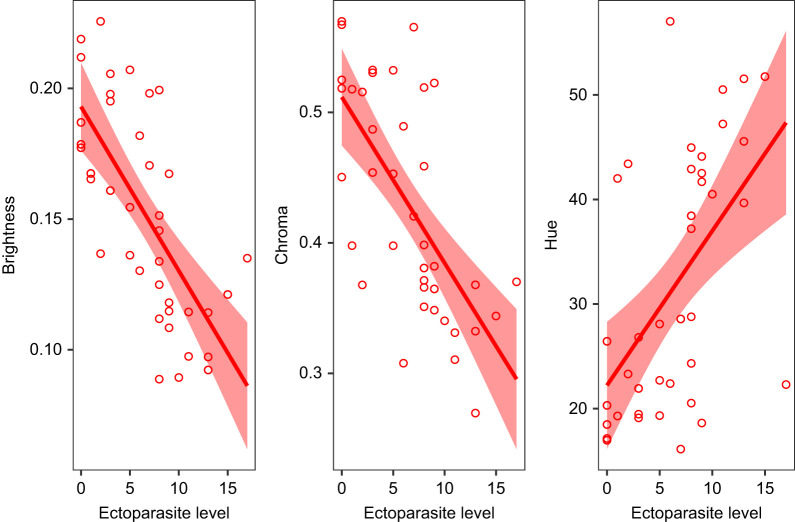
**Gular patch coloration in males varies significantly with ectoparasite level.** Lines indicate the GLMM fit between ectoparasite level (number of parasites) and brightness (χ^2^=18.36, *P*<0.001), chroma (χ^2^=16.34, *P*<0.001) and hue (χ^2^=9.24, *P*=0.002), and the shaded area constitutes the 95% confidence interval. *n=*40 males.

In males, visual signal properties such as brightness (χ^2^=18.36, *P*<0.001) and chroma (χ^2^*=*16.34, *P*<0.001) of the yellow gular patch were negatively correlated with ectoparasite level, whereas hue (χ^2^*=*9.24, *P*=0.002) was positively correlated with ectoparasite level (Fig. 2). We did not find any significant association between visual signals and body condition.

### Relationship between ectoparasite level and chemical signal

**Fig. 3.**
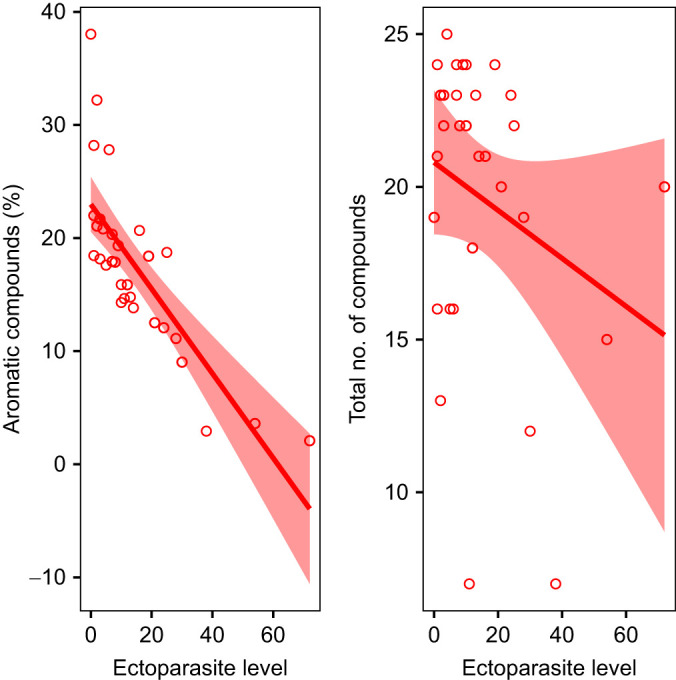
**Chemical composition of male ventral secretions varies with ectoparasite level.** Lines indicate the GLMM fit between ectoparasite level (number of parasites) and percentage aromatic compounds (χ^2^=45.02, *P*<0.001) and total number of compounds (χ^2^=0.53, *P*=0.486), and the shaded area constitutes the 95% confidence interval. *n*=30 males.

The chemical composition of femoral and pre-cloacal secretions varied among males based on their ectoparasite level. Body condition or its interaction with ectoparasite level did not show any significant effect on chemical signals. The relative proportion of aromatic compounds in male secretions was negatively associated with ectoparasite level of those males (χ^2^=45.02, *P*<0.001; Fig. 3). Specifically, benzoic acid, the only aromatic compound present in >80% of the samples, which contributed >10% of TIC, decreased in relative concentration with ectoparasite level (Fig. S3, Table S2). In contrast, the relative proportion of saturated compounds showed a positive association with male ectoparasite level (χ^2^=39.77, *P*<0.001). Among the saturated compounds, hexadecanoic acid, octadecanoic acid and heneicosane contributed to >10% of the TIC. The relative concentrations of all three compounds increased with ectoparasite level (Fig. S3, Table S2). The total number of compounds in chemical secretions of individuals was unrelated to the number of ectoparasites (χ^2^=0.53, *P*=0.468; Fig. 3). These associations between ectoparasite level and parameters of chemical secretions were still present following removal of three individuals with extremely high ectoparasite counts (>30 ectoparasites; Fig. S4).

### Female response to ectoparasite load in males: test of signal modality

**Fig. 4.**
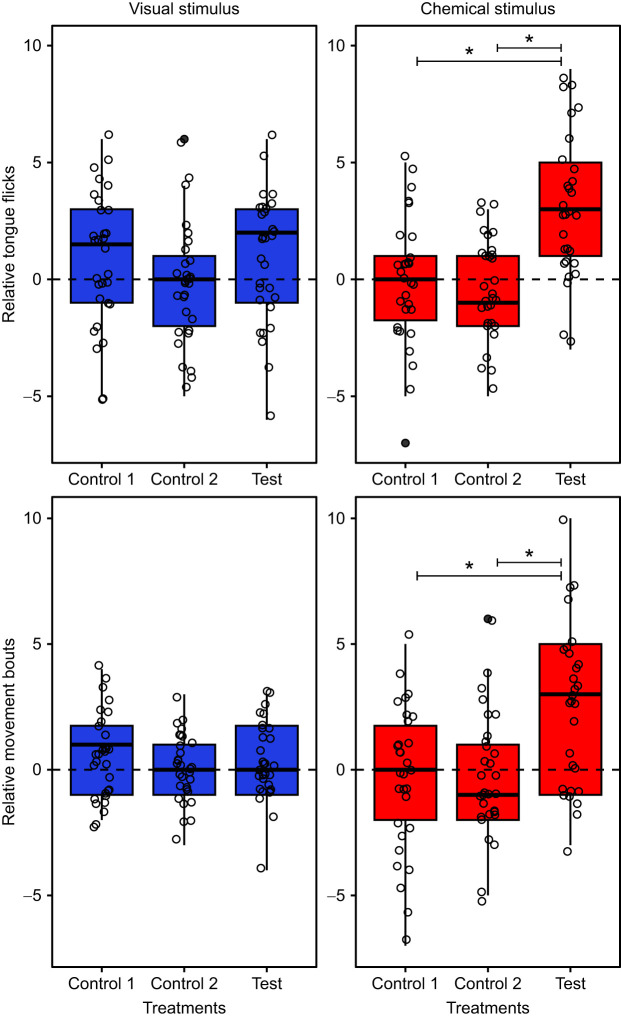
**Relative female response towards the visual or chemical traits of males presented in sequential order.** Female responses were measured as the relative number of tongue flicks or movement bouts. Trial types consisted of NP versus NP (control 1; *n=*30 trials), P versus P (control 2; *n=*30 trials) and NP versus P (test; *n=*30 trials), wherein NP denotes non-parasitised male and P denotes parasitised male. For the ‘test’ treatment, a response greater than 0 indicates a preference for NP over P male, and the inverse for values less than 0. GLMM followed by pairwise significant testing revealed that females responded with a higher number of tongue flicks and movement bouts towards NP males only using chemical signals (*P<0.05 between trial types).

To determine whether chemical or visual stimuli are used to differentiate between males of different parasite levels, females were presented with two males in sequential order and the relative behavioural response, i.e. the difference in female response between the first and the second male, was assessed. The relative number of tongue flicks did not significantly differ between control 1 (both NP males) and control 2 (both P males) in either the visual stimulus-only (*z*=−1.59, *P*=0.24) or chemical stimulus-only modalities (*z=*−0.74, *P*=0.73) (Fig. 4). Similarly, the relative number of movement bouts also did not significantly differ between control 1 and control 2 in the visual stimulus-only (*z*=−1.64, *P*=0.22) or chemical stimulus-only modalities (*z*=−0.98, *P*=0.58). In both the visual stimulus-only and chemical stimulus-only experiments, females did not show a significant preferential response for one male over the other when the two males had the same parasite level (Fig. 4).

In the test treatment, we quantified relative response by subtracting female behavioural responses towards P males from that towards NP males, wherein, a positive response would indicate higher response towards NP males. When exposed to only the visual stimulus of males under the test conditions (NP versus P), neither the relative number of tongue flicks (test versus control 1: *z*=0, *P*=1; test versus control 2: *z*=1.16, *P*=0.46) nor that of movement bouts (test versus control 1: *z*=−0.90, *P*=0.62, test versus control 2: *z*=0.55, *P*=0.84) significantly differed compared with controls*.* However, when exposed to only chemical stimulus, the relative tongue flick response (test versus control 1: *z*=3.61, *P*<0.001; test versus control 2: *z*=3.13, *P*<0.01) and relative movement bout response (test versus control 1: *z*=2.84, *P*=0.01, test versus control 2: *z*=2.75, *P*=0.02) were significantly higher in the test conditions compared with controls, indicating a higher response by females to the chemical secretions of NP males versus P males (Fig. 4).

### Female choice trials: simultaneous presentation of male stimuli

**Fig. 5.**
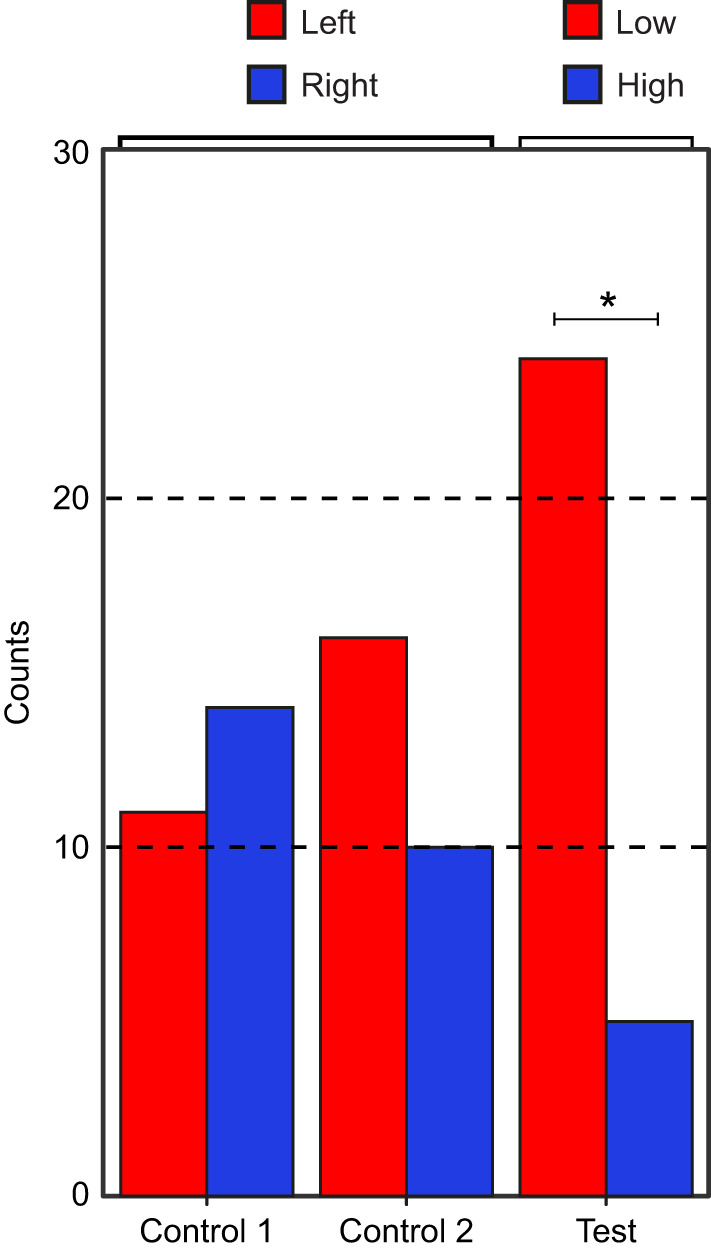
**Female choice towards the chemical traits of males presented simultaneously.** Females were offered a simultaneous choice between a low parasitised (LP) and a high parasitised (HP) male in a Y-tube setup. In control 1 (*n=*30 trials) and control 2 (*n=*30 trials), chemical secretions of two males with similar ectoparasite load (HP versus HP, LP versus LP, respectively) were presented in the left and right arm of the Y-tube. In the test (*n=*30 trials), a HP male and a LP male were presented in each arm of the Y-tube (position randomised across trials). For the two controls, coloured bars represent the choice of left or right arm of the Y-tube. For the test, red represents female approaches towards chemical signals of the LP male and blue represents female approaches to the HP male. Females approached LP males significantly more than HP males (Fisher's exact test of proportions: *z*=9.633, *P=0.002).

When given a simultaneous choice between a male with low or high ectoparasite level, females approached the chemical secretions of males with low ectoparasite levels in 24 out of 30 trials (*z=*9.633, *P*=0.002). Conversely, females randomly chose the left or right arm of the Y-tube when given a choice between two HP (*z*=0.033, *P*=0.855) or two LP males (*z*=1.633, *P*=0.201) (Fig. 5).

## DISCUSSION

In our effort to investigate the link between ectoparasite levels, male signals and female responses in *C. mysoriensis*, we found that ectoparasites not only affect individual body condition but also visual and chemical signals of males. These patterns meet a key criterion of the parasite-mediated signalling hypothesis – that secondary sexual signals can potentially convey information about male health conditions and therefore affect social and sexual signalling (Weaver et al., 2017). When testing female responses towards these signals, we found that only the chemical secretions of males with lower ectoparasite levels elicited a higher female response. Overall, although reliability of male health information is multimodal, the female response towards males is unimodal (similar to previous findings on social communication in *Cnemaspis mysoriensis*; Kabir et al., 2019).

Inter-individual variation is key for sexual signals to act as honest indicators of individual quality. In *C. mysoriensis*, variation in ectoparasite levels between individuals of both sexes was strongly correlated with body condition, which is a key health indicator. Such patterns have been recorded across vertebrates (Sánchez et al., 2018), including negative associations between ectoparasite load and body condition in other lizard species (Stuart-Fox et al., 2009). However, without experimental manipulation of ectoparasite levels, which unfortunately is difficult to do for this species, the directionality of this relationship is hard to ascertain (Sánchez et al., 2018). Parasites can aggravate health directly, by host resource depletion, as well as indirectly, through the cost of immunity (Folstad and Karter, 1992). In *C. mysoriensis*, lab observations suggest that parasitised individuals have reduced locomotor capacity, as measured by sprint speed (A.P., M.J. and M.T., unpublished data), which aligns with similar effects demonstrated in vertebrates (Setchell et al., 2009; Sorensen et al., 2016). Individuals could also vary in their ability to combat parasites (i.e. ‘good genes’ hypothesis; Zahavi, 1975), such that individuals with lower body condition are more susceptible to parasites. Regardless of the underlying mechanisms, the association between ectoparasite levels and body condition suggests negative effects of parasites on individual health.

In addition to body condition, a colour-based visual signal in males was also associated with ectoparasite level. For males, the chroma and brightness of the yellow gular region decreased while hue increased with ectoparasite level. Parasites have been found to directly and/or indirectly influence parameters of coloration, such as brightness, saturation and hue of visual signals in several lizard species (Badiane et al., 2022; Martín et al., 2008; Megía-Palma et al., 2016). For example, in *Zootoca vivipara*, ventral yellow–orange chroma decreases with mite prevalence (Badiane et al., 2022). Similarly, in *Lacerta schreiberi*, the brightness of the yellow gular patch is negatively correlated with parasite numbers while hue is positively correlated with nematodes (Megía-Palma et al., 2016). The association between gular coloration and ectoparasite levels could possibly indicate immune system quality in *C. mysoriensis*, as seen in *L. schreiberi* (Martín and López, 2009). The yellow gular patches in *C. mysoriensis* are composed of some pterins, but also carotenoids (M.T., unpublished data) that require specific dietary input and are essential in immune function (Svensson and Wong, 2011). Because of the overlap between the colour expression and immune function pathways (Koch et al., 2017), individuals capable of maintaining better physiological or cellular functioning are likely to maintain stronger immunity, as well as better visual signals (Weaver et al., 2018).

Composition, but not richness, of chemical secretions in *C. mysoriesis* males was influenced by ectoparasites. Empirical studies in other lizards have demonstrated similar links between the chemical composition of male secretions and either parasite load or immunity, or both (López and Martín, 2005; Martín and López, 2006; Martín et al., 2007). In those studies, fatty acids (e.g. ergosterol or cholesta-5,7-dien-3-ol) and aromatic compounds (e.g. benzoic acid) probably signal parasite load, and enable conspecific recognition and mate choice (Alberts, 1993). Several components of secretions are also involved in the synthesis of antioxidants, regulating the immune system or building fat reserves (Martín and López, 2015), creating a trade-off for allocation between immune function and signalling. In *C. mysoriensis* too, the major contributing components of male ventral secretions (>10% of TIC) change with ectoparasite levels. The relative concentration of benzoic acid, an aromatic compound, is lower in males with higher ectoparasite counts. By contrast, the secretions of males with higher ectoparasite levels contained higher concentrations of hexadecanoic acid, octadecanoic acid and heneicosane, which are all saturated compounds. Females of this species can, in fact, distinguish between different concentrations of specific compounds and, interestingly, the relative ratios matter less than overall concentration (Joshi et al., 2022). Because the relative proportions of both the compound types (aromatic and saturated) are inversely associated, females can potentially use either of the two to assess ectoparasite levels in males. Aromatic compounds transmit in the environment over greater distances, but several components in male secretions, such as cholesterols and saturated fatty acids, are also high molecular weight compounds, leading to low diffusion rates and longer persistence. Given that chemical signals are condition dependent and potentially costly in many species (Martín and López, 2006), the relative proportions of specific components of these secretions of male geckos could reliably indicate mate quality.

There is widespread evidence across reptiles that females choose males based on either visual (Bajer et al., 2010; Martín and López, 2009) or chemical signals (Kabir et al., 2019; Martín et al., 2007), or both (Martín and López, 2010). Multimodality in sexual signalling exists when each modality provides different information separately or more reliable information together (Møller and Pomiankowski, 1993). In other species, multiple sexual signals reliably convey information about parasite levels and health of the signaller (Martín et al., 2008), and are often used by lizards to assess signaller quality and choose mates (Bruinjé et al., 2022; Martín and López, 2006). In *C. mysoriensis*, previous experiments indicate that chemical secretions of males elicit responses by both males and females, whereas the visual signal of the coloured gular seems to only be used by other males and not females (Kabir et al., 2019). These other experiments, however, were designed to compare signal use across modalities, without considering how variation within signal components across individuals could affect communication. Here, we have established that visual and chemical trait components correlate strongly with male health status, opening up the possibility for females to assess male quality using these signals. Despite the reliability and redundancy of these signals in two modalities, our behavioural experiments indicate that females distinguish and respond to the variation in ectoparasite levels on males using only chemical secretions. When given a choice between the chemical secretions of males that vary in ectoparasite level, females not only show greater interest in the lower parasitised male through their higher number of tongue flicks and movement bouts but also approach the chemical secretions of males with lower ectoparasite load. Association preferences for ‘healthier’ males, could provide females with the direct benefit of parasite avoidance. If these preferences result in mating and if parasite resistance is heritable in this species, discriminating females would also gain indirect benefits of ‘better’ genes for their offspring (Buzatto et al., 2019).

Why is only one modality engaged in potential mate assessment when health status is encoded in multiple modalities? We speculate that the evolutionary history and ecology of this group might reveal the potential reasons for this result. The *Cnemaspis* genus made a relatively recent shift from nocturnality to diurnality (Röll, 2001), and despite variation in colour expression across species, chemical signalling remains an important modality for social communication (Kabir and Thaker, 2021). *Cnemaspis mysoriensis* are typically found in microhabitats that are dark and humid. In such environments, detection of visual signals could be difficult and unreliable, whereas chemical depositions can be detectable for longer durations even in the absence of the signaller. Signalling in the chemical modality might therefore be preferred for mate assessment because of its better suitability to the environment. Overall, our results suggest that the redundancy of information across modalities provides no signalling benefit to males, as females remain reliant on chemical-based secondary sexual characters to assess male quality.

## Supplementary Material

10.1242/jexbio.246217_sup1 Supplementary information
