# Supplementary material for: Too much information? Males convey parasite levels using more signal modalities than females utilise
Source: J Exp Biol. 2024 Jan 10;227(1):jeb246217. doi: 10.1242/jeb.246217 (PMC10906484; doi:10.1242/jeb.246217)
Supplement: Supplementary information [file jexbio-227-246217-s1.pdf]

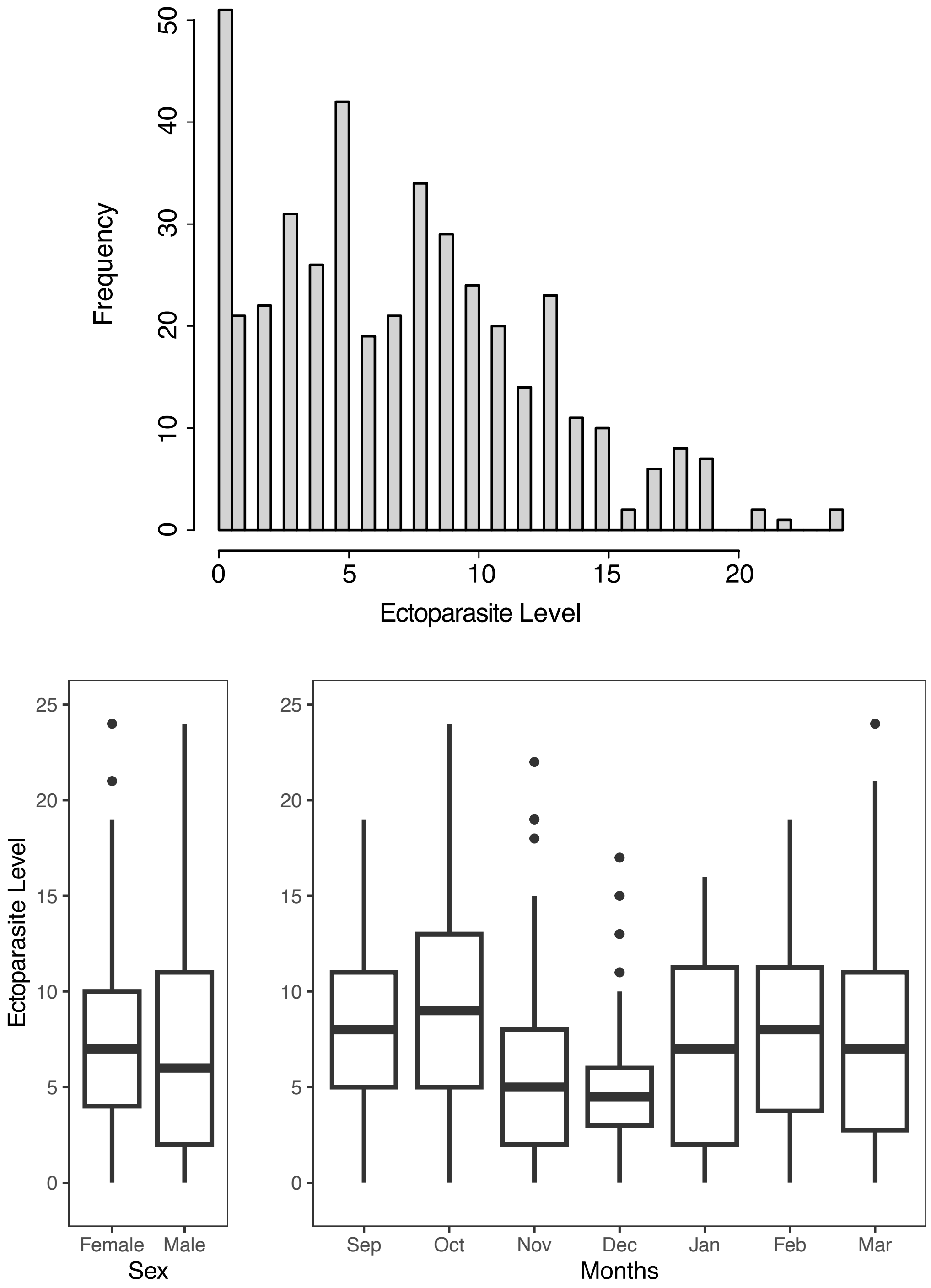

Fig. S1. Top – Distribution of ectoparasite levels. Shapiro-wilk normality test suggests the distribution is non-normal ($p < 0.001$). **Bottom** – Variation in ectoparasite levels across sampling months. Ectoparasite levels did not significantly vary across sex but varied across some of the sampling months, with significantly lower levels of parasites found during the cooler and wetter months of November and December compared to the rest of the months.

Journal of Experimental Biology • Supplementary information

A Female response trial with ***sequential*** exposure to male signals

Signal Modality	Control 1		Control 2		Test	
	**Exposure 1**	**Exposure 2**	**Exposure 1**	**Exposure 2**	**Exposure 1***	**Exposure 2***
Visual only	NP male 1 only	NP male 2 only	P male 1 only	P male 2 only	NP male only	P male only
Chemical only	NP male 1 chemicals only	NP male 2 chemicals only	P male 1 chemicals only	P male 2 chemicals only	NP male chemicals only	P male chemicals only

** exposure order randomized*

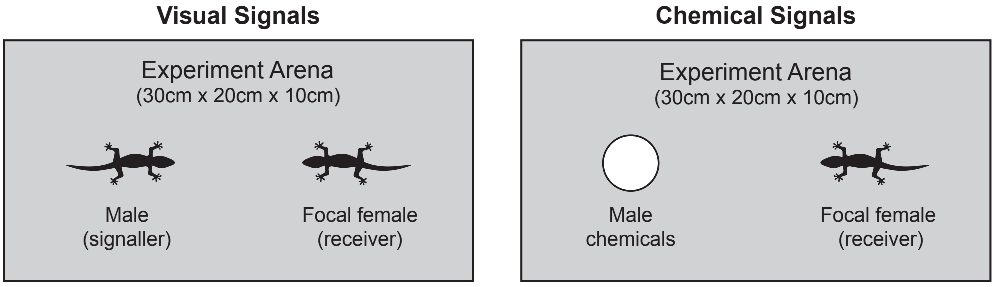

B Female choice trial with ***simultaneous*** exposure to male signals

Signal Modality	Control 1	Control 2	Test
Chemical only	2 LP male chemicals only	2 HP male chemicals only	1 LP and 1 HP male

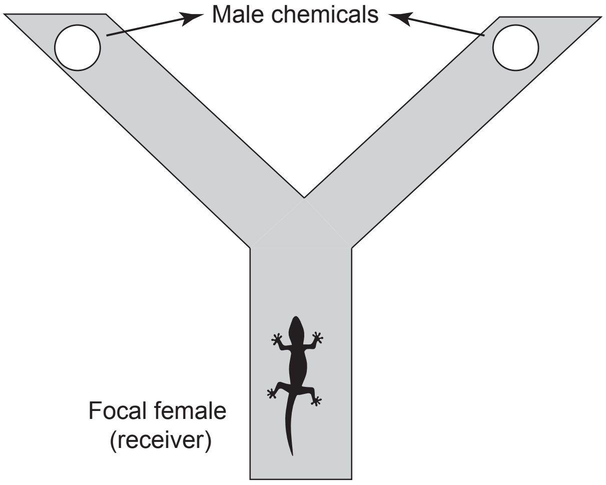

Fig. S2. Schematic for behavioural trials where focal females were either (A) *sequentially* or (B) *simultaneously* exposed to male signal stimuli. P: parasitised male; NP: non-parasitised male; LP: Low-parasitised male; HP: High-parasitised male.

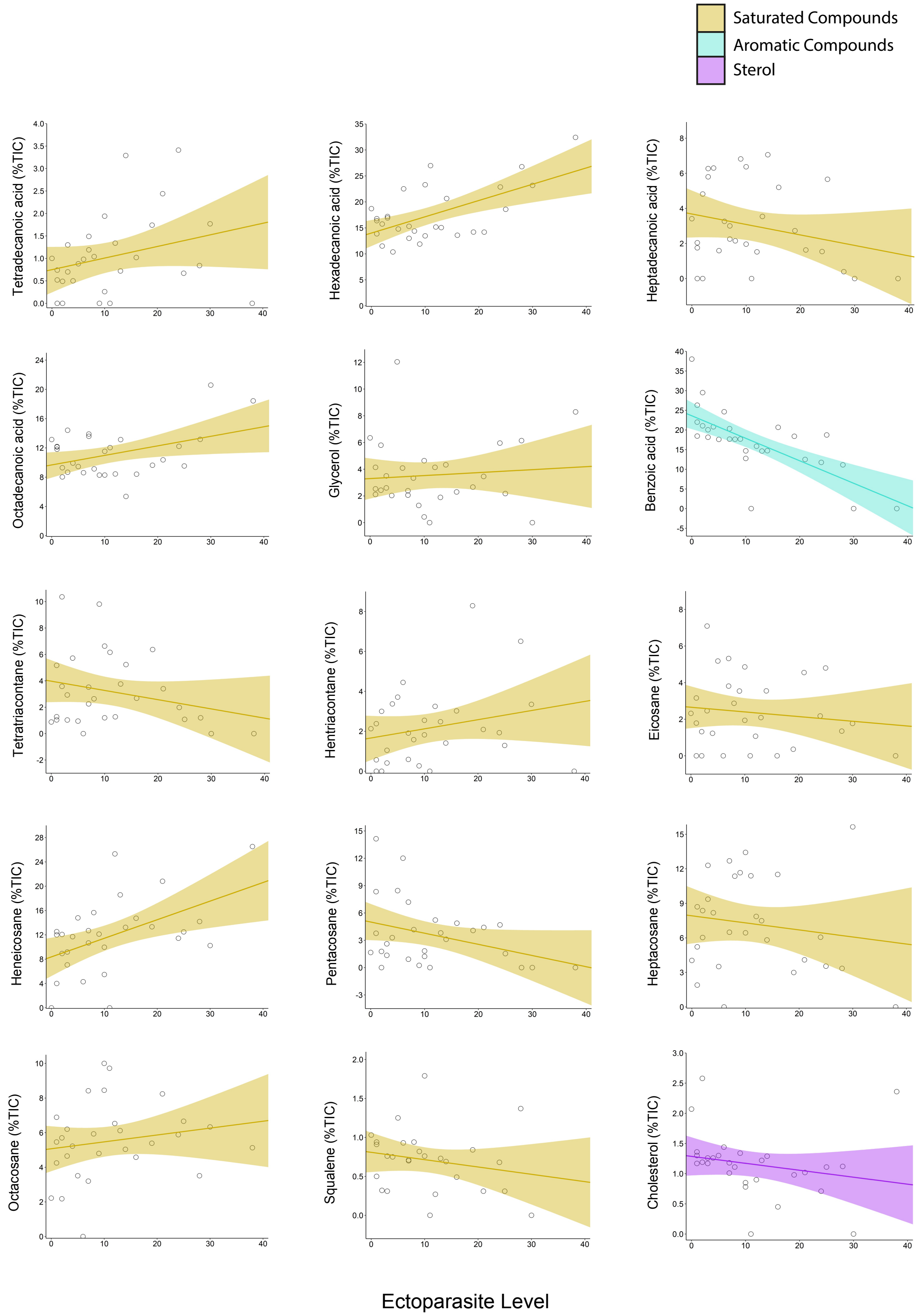

Fig. S3. Relative proportions (%TIC) of each of the chemical compounds in ventral secretions of males with different ectoparasite levels. The lines indicate the linear fit, while the shaded area constitutes 95% confidence interval.

Journal of Experimental Biology • Supplementary information

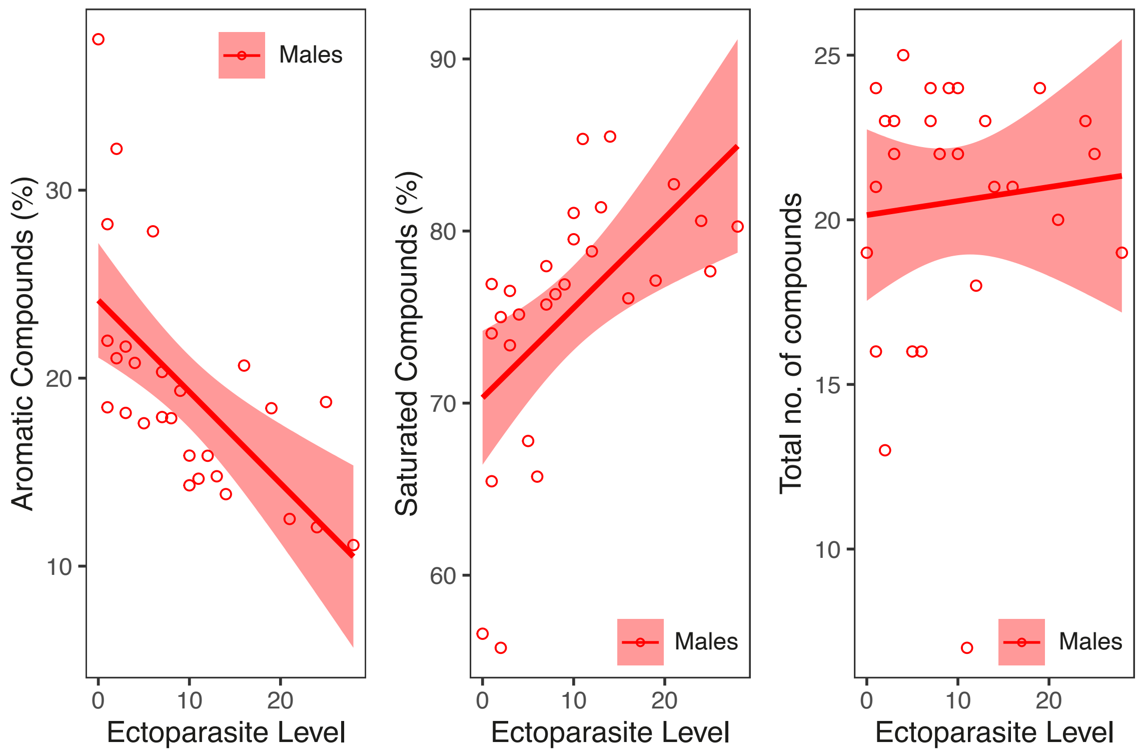

Fig. S4. Chemical composition in ventral secretions for males with less than 30 ectoparasites vary with their ectoparasite level. Lines indicate GLM fit, wherein the shaded area constitutes the 95% confidence interval.

Table S1.

**Coefficients:**	**Estimate**	**Std. Error**	**z value**	**Pr(>|z|)**	
(Intercept)	4.01	0.13177	30.38	< 2e-16	***
Lizards: Males	-0.22	0.16045	-1.4	0.16208	
Body Condition	-5.26	0.34895	-15.08	< 2e-16	***
Month: Oct	0.043	0.06212	0.71	0.47983	
Month: Nov	-0.18	0.07171	-2.56	0.01034	*
Month: Dec	-0.45	0.07592	-5.94	2.80e-09	***
Month: Jan	-0.02	0.06756	-0.3	0.76723	
Month: Feb	-0.01	0.06526	-0.02	0.9802	
Month: Mar	-0.01	0.06441	-0.2	0.84055	
Lizards: Males x Body Condition	1.61	0.42342	3.82	0.00014	***

Estimated regression parameters, standard errors, *z*-values and *p*-values for the Poisson GLMM to model relationship of ectoparasite level with body condition (measured as scaled mass index), sex and sampling months. Estimated variance of the random intercept: *plot* is 0.004264, and standard deviation is 0.0653. Number of observations: total = 426, plots = 5. AIC = 2259.7, Log-likelihood = –1117.86. Significance codes: *** < 0.001; 0.001 < ** < 0.01; 0.01 < * < 0.05.

Table S2.

Sr. no.	Compound	Category	%TIC ± SE
1	Hexadecanoic acid	Saturated	18.13 ± 0.99
2	Benzoic acid	Aromatic	15.99 ± 1.6
3	Heneicosane	Saturated	11.86 ± 1.09
4	Octadecanoic acid	Saturated	11.07 ± 0.65
5	Heptacosane	Saturated	7.10 ± 0.74
6	Octacosane	Saturated	5.65 ± 0.42
7	Pentacosane	Saturated	3.40 ± 0.64
8	Glycerol	Saturated	3.33 ± 0.47
9	Heptadecanoic acid	Saturated	3.03 ± 0.41
10	Tetratriacontane	Saturated	2.97 ± 0.5
11	Eicosane	Saturated	2.24 ± 0.35
12	Hentriacontane	Saturated	2.20 ± 0.33
13	Tetradecanoic acid	Saturated	1.97 ± 0.81
14	Cholesterol	Sterol	1.18 ± 0.12
15	Squalene	Saturated	0.98 ± 0.18

Chemical compounds in the ventral secretions of males. Compound names and their respective mean percentage total ionic count (%TIC ± SE) are shown here.

Journal of Experimental Biology • Supplementary information
